# Dynamic Variations in Brain Glycogen are Involved in Modulating Isoflurane Anesthesia in Mice

**DOI:** 10.1007/s12264-020-00587-3

**Published:** 2020-10-13

**Authors:** Ze Fan, Zhihao Zhang, Shiyi Zhao, Yuanyuan Zhu, Dong Guo, Bo Yang, Lixia Zhuo, Jiao Han, Rui Wang, Zongping Fang, Hailong Dong, Yan Li, Lize Xiong

**Affiliations:** 1 Department of Anesthesiology and Perioperative Medicine, Xijing Hospital, Fourth Military Medical University, Xi’an, 710032 Shaanxi China; 2 College of Basic Medicine, Fourth Military Medical University, Xi’an, 710032 Shaanxi China; 3 Department of Neurobiology, Fourth Military Medical University, Xi’an, 710032 Shaanxi China; 4 Center for Brain Science and Department of Anesthesiology, The First Affiliated Hospital of Xi’an Jiaotong University, Xi’an, 710061 Shaanxi China; 5 Translational Research Institute of Brain and Brain-Like Intelligence, Department of Anesthesiology and Perioperative Medicine, Shanghai Fourth People’s Hospital Affiliated to Tongji University School of Medicine, Shanghai, 200081 China

**Keywords:** Anesthesia-arousal, Brain glycogen, General anesthesia, Glycogen phosphorylase, Glycogen synthetase, Isoflurane

## Abstract

General anesthesia severely affects the metabolites in the brain. Glycogen, principally stored in astrocytes and providing the short-term delivery of substrates to neurons, has been implicated as an affected molecule. However, whether glycogen plays a pivotal role in modulating anesthesia–arousal remains unclear. Here, we demonstrated that isoflurane-anesthetized mice exhibited dynamic changes in the glycogen levels in various brain regions. Glycogen synthase (GS) and glycogen phosphorylase (GP), key enzymes of glycogen metabolism, showed increased activity after isoflurane exposure. Upon blocking glycogenolysis with 1,4-dideoxy-1,4-imino-D-arabinitol (DAB), a GP antagonist, we found a prolonged time of emergence from anesthesia and an enhanced δ frequency in the EEG (electroencephalogram). In addition, augmented expression of glycogenolysis genes in glycogen phosphorylase, brain (Pygb) knock-in (Pygb^H11/H11^) mice resulted in delayed induction of anesthesia, a shortened emergence time, and a lower ratio of EEG-δ. Our findings revealed a role of brain glycogen in regulating anesthesia–arousal, providing a potential target for modulating anesthesia.

**Electronic supplementary material:**

The online version of this article (10.1007/s12264-020-00587-3) contains supplementary material, which is available to authorized users.

## **Introduction**

General anesthetics are widely used clinically. However, the mechanisms through which general anesthetics induce quiescent neuronal activity and cause unconsciousness remain largely unknown [1–6]. In recent decades, the role of cerebral energy metabolism has attracted increasing attention [7, 8]. It has been revealed that mutations in complex I of mitochondria increase the sensitivity of *Caenorhabditis elegans* to volatile anesthetics [9–12]. Recently, emerging evidence has indicated that a reduction in cellular adenosine triphosphate (ATP) levels is associated with a delay in isoflurane-induced loss of the righting reflex (LORR) in mice [13], suggesting that changes in available cerebral energy might regulate anesthetic effects [11–13].

Brain glycogen, principally stored in astrocytes rather than in neurons, is considered to be the largest on-demand energy source in the brain [14–17]. Glycogen can be metabolized into phosphorylated glucose and subsequently produce pyruvate, which is eventually converted to lactate [15]. According to the “lactate shuttle” hypothesis, the lactate derived from astrocytic glycogen is transferred into neighboring neurons to fuel the tricarboxylic acid cycle [18–20]. In addition, a growing body of evidence indicates that astrocytic glycogenolysis and the subsequent lactate transport play a crucial role in memory formation, long-term potentiation of synapses, and other physiological and pathological events in the brain [17, 21–23]. Although general anesthetics such as ether and pentobarbital have been reported to induce increased levels of brain glycogen [24–26], dynamic changes in brain glycogen throughout anesthesia and their association with anesthetic effects remain unclear. Moreover, since the amount of brain glycogen is steadily and dynamically maintained due to the balance between glycogenesis and glycogenolysis, two key enzymes – glycogen synthase (GS) and glycogen phosphorylase (GP) – are responsible for the regulation of brain glycogen levels [15]. However, much less attention has been directed toward the effects of anesthetic agents on glycogen metabolic enzymes.

In this study, we investigated changes in the brain glycogen levels and key enzymes of glycogen metabolism in the cortex, hippocampus, thalamus, and striatum during isoflurane anesthesia in mice. Furthermore, an inhibitor of GP (DAB) and transgenic Pygb knock-in (Pygb^H11/H11^) mice were used to test the hypothesis that brain glycogen plays a crucial role in modulating anesthesia–arousal.

## **Materials and Methods**

### Animals and Experimental Protocols

Male C57BL/6 mice (8 weeks old) weighing 22 g–25 g were purchased from the Animal Experimental Center of the Fourth Military Medical University (Xi’an, China), and all experiments were conducted with the approval of the Ethics Committee for Animal Experimentation of the Fourth Military Medical University. Mice were housed in an environment with a temperature of 22°C–24°C, humidity 38%–42%, and a light-dark cycle of 12 h/12 h. Standard rodent chow and tap water were available *ad libitum*. Mice with Pygb knocked in at the H11 locus on chromosome 11 (heterozygous: Pygb^H11/+^; from Cyagen Biosciences Inc., Guangzhou, China) were housed in a specific pathogen-free environment and were interbred to obtain homozygous Pygb^H11/H11^ mice and wild-type (WT) littermates.

**Experiment 1:** To investigate whether brain glycogen levels change during isoflurane anesthesia, 12 mice were randomly divided into two groups (*n* = 6 per group): a control group and an isoflurane exposure group (2 h of exposure). Then, the levels of brain glycogen in the cortex (CTX), hippocampus (HIPPO), thalamus (THAL) and striatum (STRIAT) were measured. To further evaluate the dynamic variations in brain glycogen during isoflurane anesthesia, the glycogen levels in the mice exposed to 1.4% isoflurane for different durations (0.5, 1, 2, or 4 h; *n* = 6 per duration) and in control mice were measured. We also assessed the glycogen levels under both control conditions and the following anesthetic states: LORR, isoflurane exposure for 2 h, recovery of righting reflex (RORR), and 2 h after isoflurane exposure (*n =* 8 per group).

**Experiment 2:** The effects of isoflurane exposure on key glycogen metabolic enzymes were examined. 8 mice were randomly divided into two groups (*n =* 4 per group): a control group and an isoflurane exposure group (2 h of exposure). The levels of phosphorylated GS and GP and their activities were measured in the indicated brain regions as described previously.

**Experiment 3:** To determine the role of brain glycogen in the induction of, maintenance of, and recovery from isoflurane anesthesia, 16 mice were randomly divided into two groups (*n =* 8 per group): intracerebroventricular injection of DAB (2 μL, 300 pmol) 15 min before isoflurane exposure and a saline injection group [23, 27]. Pygb^H11/H11^ mice and WT littermates were also used (*n =* 8 per group). For these mice, the time to LORR and to RORR under 1.4% isoflurane exposure as well as the total percentage of power in the EEG under 0.8% isoflurane exposure were recorded.

### Isoflurane Exposure

Mice were exposed to 1.4% isoflurane (R510-22, RWD Life Science, Shenzhen, China) in 98.6% oxygen (oxygen flow, 1 L/min) in a chamber with an air intake and exhaust system (20×10×11 cm^3^). The temperature was controlled at 37°C during anesthesia. The oxygen, carbon dioxide, and isoflurane levels were continuously monitored (MP-60, Phillips Medical Systems, Best, The Netherlands), and post-anesthesia care was carried out. All instances of isoflurane exposure were performed at 08:00–12:00 to avoid the influence of circadian rhythms. The control mice were administered 100% oxygen.

### Brain Tissue Preparation and Glycogen Measurement

A focused microwave irradiation system (ORW1.5S-Focus, Orient Microwave, Nanjing, China) was used to rapidly fix brain tissue and prevent glycogen breakdown [28]. Briefly, a mouse was confined in a specialized animal containment device. A high-energy microwave (1 kW) was focused on the head for 5 s, after which the brain was immediately removed and different regions were dissected under a stereomicroscope (SZ51, Olympus, Tokyo, Japan). For staining experiments, the brain was fixed in and incubated with 4% paraformaldehyde prior to paraffinization and sectioning. The glycogen levels in these samples were assessed with a glycogen assay kit (K648, BioVision, Milpitas, USA) or a periodic acid-Schiff (PAS) staining kit (ab150680, Abcam, Cambridge, MA, USA).

### Enzyme Activity Assay

GS and GP activities were determined with assay kits (GMS50500.2 for GS and GMS50092.2 for GP, Genmed Scientifics, Shanghai, China). Relative activity was calculated by measuring the absorbance at 340 nm on a microplate reader (infinite M200, Tecan, Männedorf, Switzerland) and then by normalizing the value to that of the total protein levels in similar samples.

### Immunoblotting

The brain tissue of mice was homogenized in RIPA lysis buffer (Beyotime, Nantong, China) containing a complete protease inhibitor and phosphatase inhibitor cocktail (1%, Thermo Fisher Scientific, Waltham, MA, USA). The protein concentration was determined with a BCA protein assay kit (23225, Thermo Fisher Scientific). Equivalent amounts of protein (30 µg per lane) were loaded and separated on SDS-PAGE gels and then transferred to 0.22-μm PVDF membranes (10600021, GE Healthcare, Munich, Germany) for 2 h. After the membranes were blocked with 5% skim milk, they were incubated overnight at 4°C with the following primary rabbit antibodies (Table S1): anti-PYGB (ab154969, 1:1000, Abcam), anti-GYS1 (ab40867, 1:1000, Abcam), anti-pSer641-GS (3891, 1:1000, CST), anti-pSer15-PYGB (synthesized by Genecreate Biological Engineering Co., Ltd, Wuhan, China), anti-AGL (ab133720, 1:1000, Abcam), anti-GBE1 (ab180596, 1:1000, Abcam), and anti-β-actin (ab119716, 1:1000, Abcam). After three washes, the membranes were incubated with goat anti-rabbit secondary antibody conjugated to horseradish peroxidase (ab6721, 1:5000, Abcam) for 2 h at room temperature. The membranes were washed again, and the bands were detected using a chemiluminescent horseradish peroxidase substrate (1812401, Millipore, MA, USA). The optical density of each band was analyzed using Quantity One software 5.0 (Bio-Rad, La Jolla, CA, USA).

### Immunohistochemical/Immunofluorescent Staining

After brain sections were deparaffinized, endogenous peroxidase activity was blocked by immersion in hydrogen peroxide for 10 min. Then, the sections were incubated with 5% normal donkey serum for 1 h to block non-specific binding. Subsequently, the sections were incubated at 4°C overnight with the following primary antibodies as appropriate (Table S1): rabbit anti-PYGB (HPA031067, 1:50, Atlas), rabbit anti-GYS1 (1:25, Abcam), mouse anti-NeuN (MAB377, 1:200, Millipore), chicken anti-GFAP (GTX85454, 1:500, GeneTex), and goat anti-Iba1 (ab5076, 1:300, Abcam). The sections were then treated with fluorescent secondary antibodies as follows: AlexaFluor 488 (green)-conjugated goat anti-rabbit (A32731, 1:500), AlexaFluor 594 (red)-conjugated goat anti-chicken (A32759, 1:500), AlexaFluor 594-conjugated donkey anti-mouse (A32744, 1:500), and AlexaFluor 594-conjugated donkey anti-goat (A11058, 1:500) (all from Thermo Fisher Scientific). Nuclei were stained with DAPI (4’,6-diamidino-2-phenylindole; 1:1000; Sigma-Aldrich, St Louis, MO, USA). Biotinylated secondary antibody (ab6721, 1:5000, Abcam) and peroxidase-conjugated streptavidin followed by chromogenic reaction with 3,3’-diaminobenzidine were added to sections for immunohistochemical staining, which were counterstained with hematoxylin.

### Blood Glucose Analysis

The blood glucose levels were determined using a blood glucose meter (OneTouch Ultra, Johnson& Johnson, New Brunswick, NJ, USA).

### Intracerebroventricular Injection

DAB (2 μL, 300 pmol, D1542, Sigma-Aldrich) or saline was injected into the lateral ventricle through a cannula (62003, RWD Life Science) placed 1 week before the experiment (0.22 mm posterior to bregma, 1.0 mm lateral, 1.5 mm deep).

### Analysis of Anesthesia Induction and Emergence

Each mouse was placed in a rotating cylinder and then exposed to 1.4% isoflurane. The induction time was defined as the interval from the beginning of anesthesia to the LORR. Similarly, the emergence time was defined as the interval from the termination of anesthesia to the RORR.

### EEG Recording and Analysis

For EEG recording, electrodes were implanted 7 days before experiments. After 0.8% isoflurane exposure for 30 min, EEG signals were digitized at 1 kHz by a PowerLab and LabChart system (AD Instruments, Dunedin, New Zealand) and were bandpass filtered at 0.3 Hz–50 Hz. The analysis of the percentage total power and construction of spectra were completed in MatLab (MathWorks, Natick, MA). EEG changes in the frequency bands δ (0.3 Hz–4 Hz), θ (4 Hz–10 Hz), α (10 Hz–15 Hz), β (15 Hz–25 Hz), and γ (25 Hz–50 Hz) were used to estimate the wake/anesthesia states.

### Statistical Analysis

GraphPad Prism 7.0 (GraphPad Software, San Diego, CA) was used for statistical analysis. All results are shown as the mean ± SEM. Comparisons between two groups were made using an unpaired *t*-test, and comparisons between multiple groups were made using one-way ANOVA with Tukey-Kramer’s *post hoc* test. To determine statistically significant differences among groups at different time points throughout anesthesia delivery, one-way ANOVA with Sidak’s multiple comparisons was used. *P <* 0.05 was considered statistically significant.

## **Results**

### Elevated Brain Glycogen Levels After Isoflurane Exposure

**Fig. 1:**
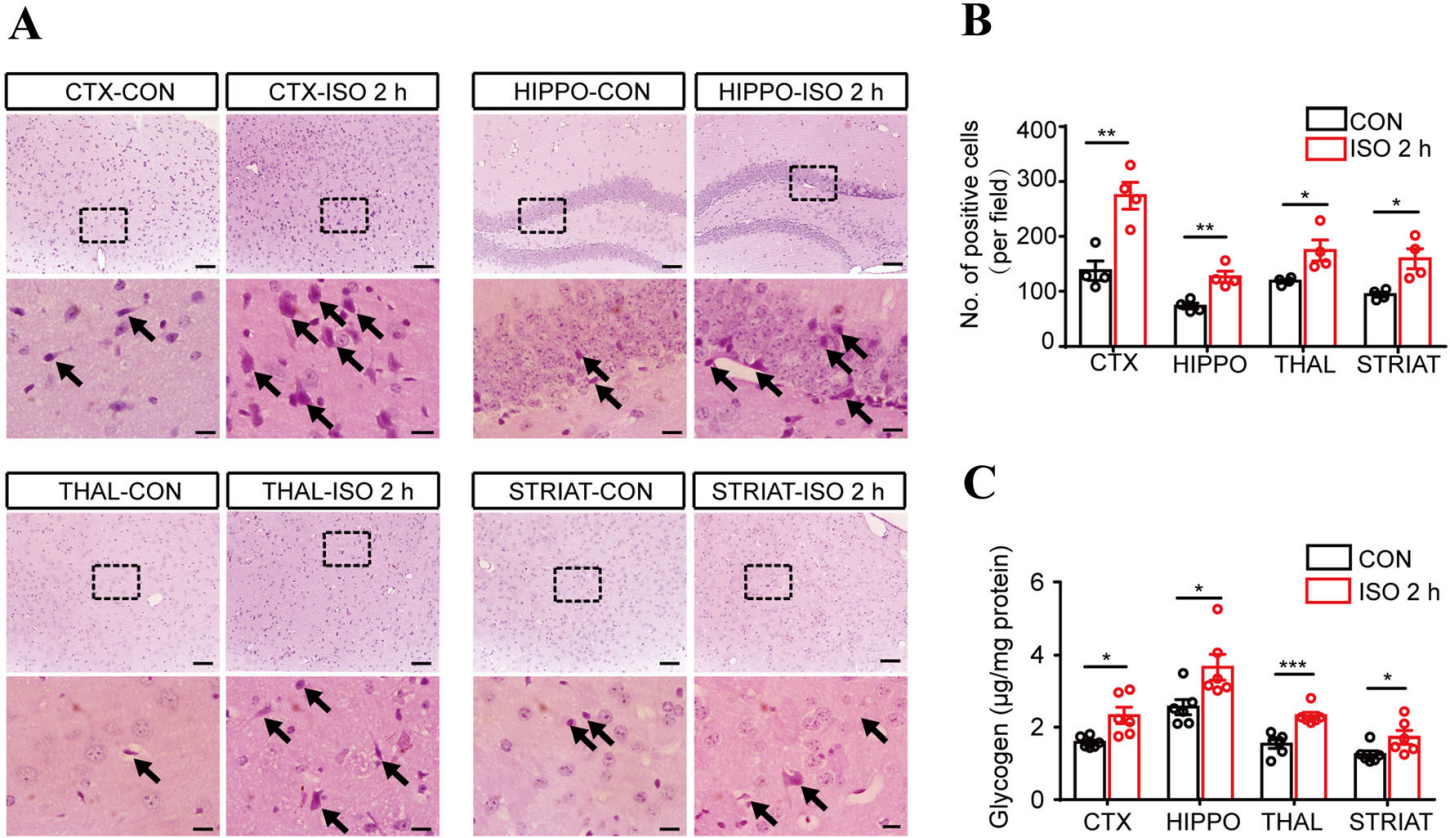
Brain glycogen levels increase in mice under isoflurane anesthesia. **A** Representative PAS staining of sections in the four brain regions. (arrows, cells with abundant glycogen; scale bars, 100 μm in upper panels, 20 μm in lower panels). **B** Numbers of PAS-positive cells per field under 20× magnification (*n* = 4 per group). **C** Glycogen levels (*n* = 6 per group). *P < 0.05, **P < 0.01, ***P < 0.001 *vs* control. CON, oxygen control; ISO 2 h, isoflurane exposure for 2 h; CTX, cortex; HIPPO, hippocampus; THAL, thalamus; STRIAT, striatum.

Mice were exposed to 1.4% isoflurane in 98.6% oxygen. Glycogen levels were measured in the CTX, HIPPO, THAL, and STRIAT. PAS staining revealed that after isoflurane exposure for 2 h, more intense signals were observed in the analyzed regions compared to those in the control group (Fig. 1A, B). Consistent with these data, the glycogen assay kit revealed higher glycogen levels in the homogenates of the CTX, HIPPO, THAL, and STRIAT after 2 h of isoflurane exposure (Fig. 1C). These data suggested that isoflurane anesthesia increases the glycogen levels in various brain regions, including the CTX, HIPPO, THAL, and STRIAT.

### Dynamic Variations in Glycogen Levels Under Isoflurane Anesthesia

**Fig. 2:**
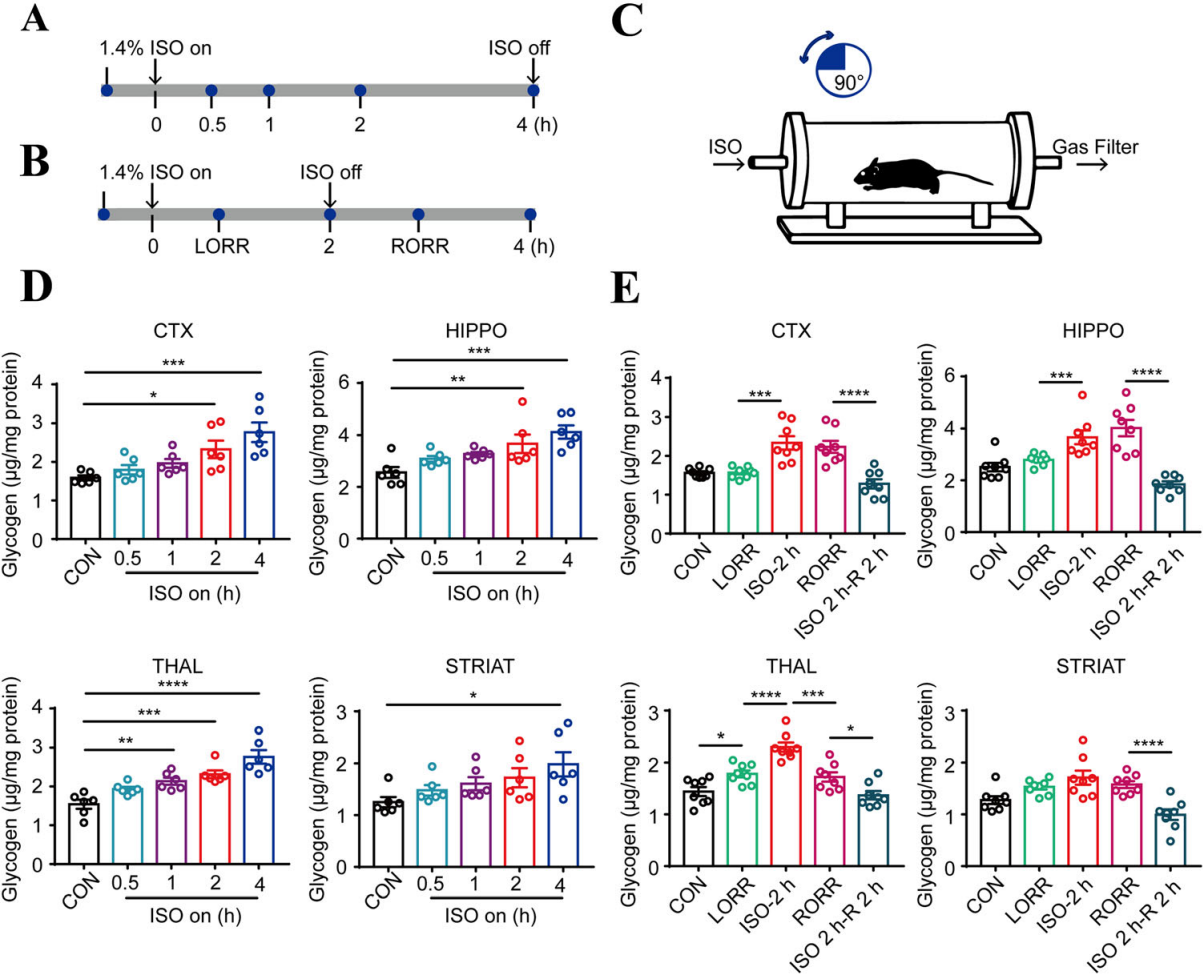
Dynamic variations in glycogen levels in mice under isoflurane anesthesia. **A** Schematic of the different time points at which brain glycogen levels during administration of isoflurane anesthesia were measured. **B** Schematic of brain glycogen at key time points during isoflurane anesthesia. **C** Cartoon of observation of righting reflex behavior. **D** Brain glycogen slowly accumulates in a time-dependent manner in different brain regions (*n =* 6 per group). **E** Patterns of regional changes in brain glycogen during isoflurane anesthesia (*n =* 8 per group). *P < 0.05, **P < 0.01, ***P < 0.001, and ****P < 0.0001. ISO 2 h-R 2 h, 2 h after isoflurane exposure; LORR, loss of righting reflex; RORR, recovery of righting reflex.

Next, we evaluated the temporal changes in glycogen levels during extended exposure to isoflurane (Fig. 2A). We noted that the degree of glycogen increase in the four regions was positively related to the duration of isoflurane anesthesia (Fig. 2D). We then assessed the glycogen levels under different anesthetic states according to the righting reflex behavior: no anesthesia (control), LORR, isoflurane exposure for 2 h, RORR, and 2 h after isoflurane exposure (Fig. 2B, C). Generally, in the analyzed regions, the glycogen levels were elevated during anesthesia but reduced during arousal. Notably, the THAL displayed remarkable sensitivity to changes in anesthetic state because each state showed significant differences in glycogen levels in the THAL, while other regions did not show this pattern (Fig. 2E). Collectively, these results revealed a changing pattern of glycogen in specific brain regions under isoflurane anesthesia.

### Glycogen Metabolism is Activated After Isoflurane Exposure

**Fig. 3:**
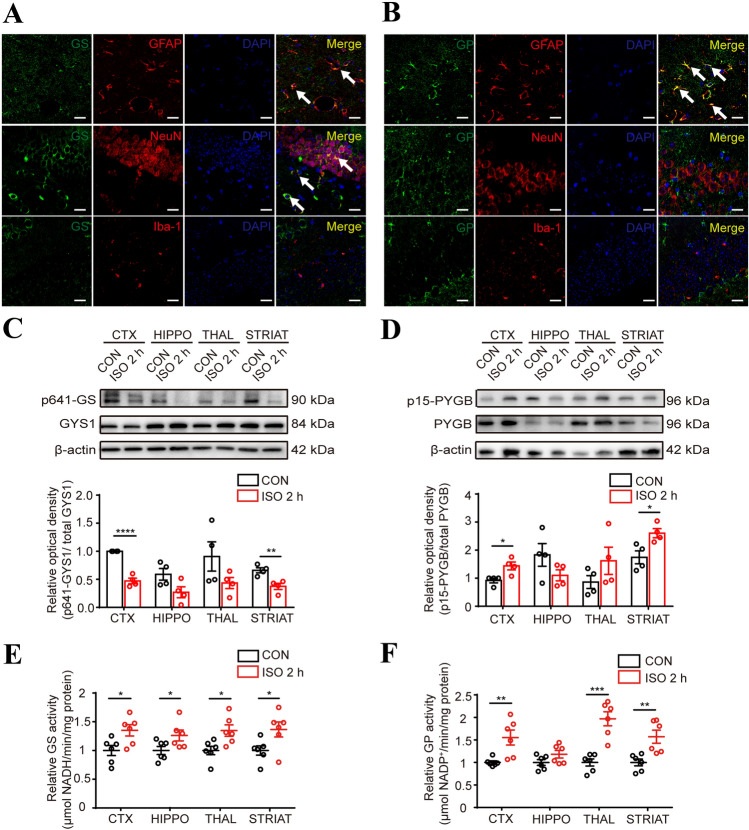
Effects of isoflurane on enzymes related to glycogen metabolism. **A**, **B** Representative images of immunofluorescent staining of GS (**A**) and GP (**B**) with GFAP, NeuN and Iba-1 (arrows, co-labeled cells; scale bars, 20 μm). **C**, **D** Levels of phosphorylated GS at Ser641 (**C**) and phosphorylated GP at Ser15 (**D**) (*n =* 4 per group). **E**, **F** Activity of GS (**E**) and GP (**F**) in the presence or absence of isoflurane exposure (*n =* 6 per group). *P < 0.05, **P < 0.01, ***P < 0.001, ****P < 0.0001 *vs* control.

As glycogen levels are maintained in a balance between glycogenesis and glycogenolysis, we examined the key enzymes of glycogen metabolism. First, we used immunofluorescence to localize GS and GP. We found that GP was predominately co-localized with GFAP rather than with NeuN or Iba1, and GS co-localized with both GFAP and NeuN (Fig. 3A, B). These results indicated that both neurons and astrocytes are able to synthesize glycogen. However, most relevant to the role of brain glycogen as an energy source is the putative role of astrocytes in regulating glycogenolysis. Next, to verify the effects of isoflurane anesthesia on glycogen metabolism, we assessed the expression of key enzymes in the CTX, HIPPO, THAL and STRIAT. No significant differences were found after isoflurane exposure for 2 h (Fig. S1). However, the level of phosphorylated GS at Ser641 (p641-GS), which represents the inactivated form, was lower in various brain regions in the isoflurane-treated group than that in the control group, although there were no significant differences in these levels in the HIPPO and THAL (Fig. 3C). Concurrently, increased GS activity was also found (Fig. 3E). Similarly, the levels of phosphorylated GP at Ser15 (p15-GP) showed increased trends in the CTX, THAL, and STRIAT (Fig. 3D), which was consistent with the increased GP activity in these regions (Fig. 3F). Notably, GP activity and the phosphorylation level in the HIPPO showed no significant differences between the two groups (Fig. 3D, F). Therefore, these results demonstrated that isoflurane exposure enhances both the anabolism and catabolism of brain glycogen, although there is heterogeneity among regions.

### Inhibition of Glycogenolysis Enhances EEG-δ Frequency and Delays Emergence from Anesthesia

**Fig. 4:**
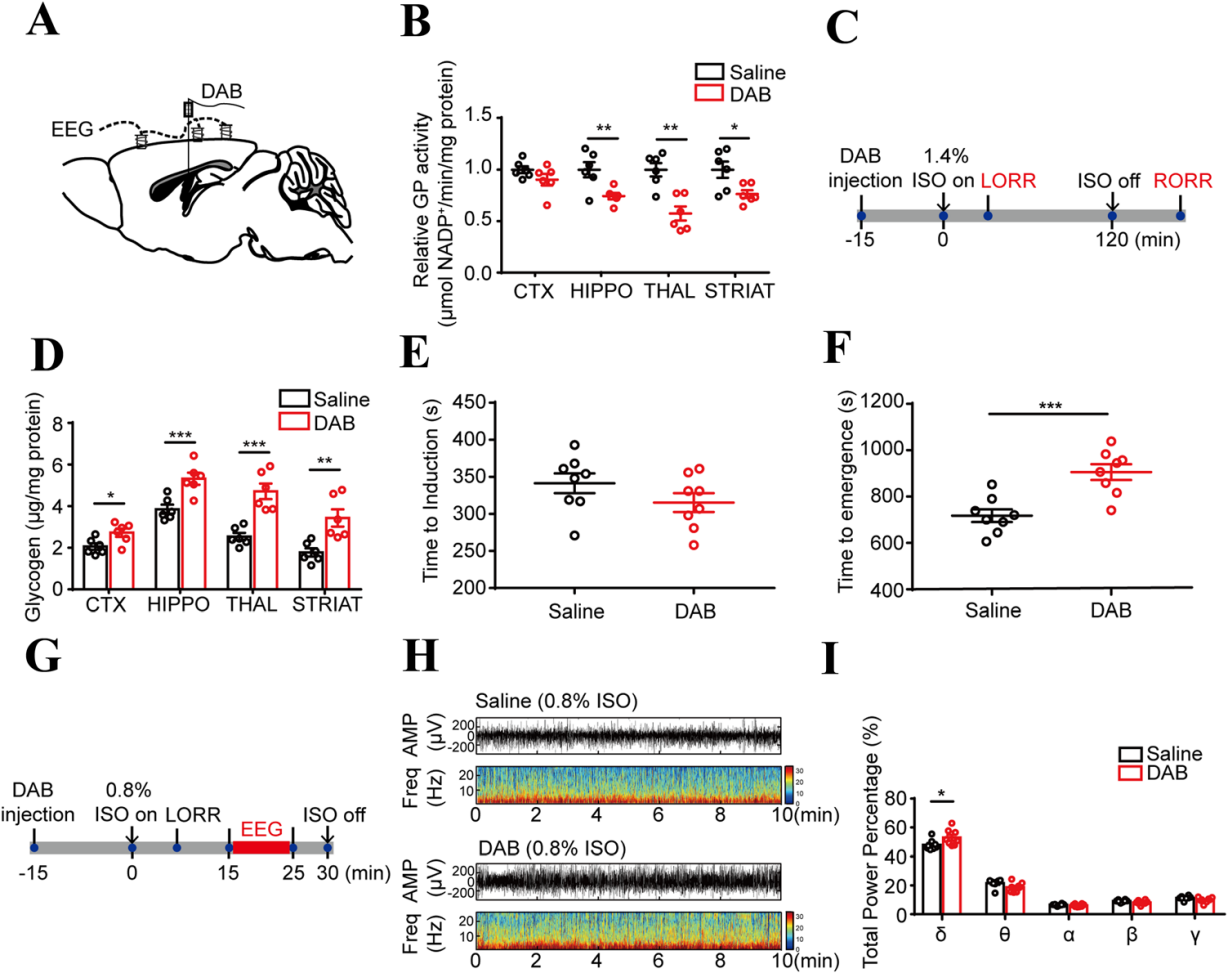
Inhibiting glycogenolysis with DAB accelerates induction of isoflurane anesthesia in mice. **A** Diagram of the intracerebroventricular injection site. **B** GP activity in the brain regions 2 h after DAB injection (*n* = 6 per group). C Protocol for recording the LORR and RORR times of mice under 1.4% isoflurane anesthesia. **D** Brain glycogen levels in the selected regions under isoflurane anesthesia for 2 h after DAB or saline injection (*n* = 6 per group). **E**. **F** The LORR (**E**) and RORR (**F**) times in the DAB and saline injection groups after 2 h of isoflurane exposure (*n* = 8 per group). **G** Protocol for recording the EEG in mice under 0.8% isoflurane anesthesia. EEG recording for 10 min began at 15 min after initial administration of isoflurane. **H** Representative EEG traces and heat maps. (I) Total percentage power of the EEG in mice under 0.8% isoflurane exposure (*n* = 8 per group). *P < 0.05, **P < 0.01, ***P < 0.001.

To explore whether the variations in brain glycogen affect the isoflurane-induced EEG spectrum and anesthesia–arousal, DAB (2 μL, 300 pmol), a GP antagonist, was injected into the lateral ventricle (Fig. 4A). DAB injection led to a significant decrease in GP activity in the HIPPO, THAL, and STRIAT (Fig. 4B). GP activity in the CTX was also down-regulated, but there were no significant differences between the DAB and saline groups (Fig. 4B). At 15 min after DAB administration, mice were exposed to isoflurane (Fig. 4C). The glycogen levels were higher in the DAB injection group than in the saline group after isoflurane exposure for 2 h (Fig. 4D). Despite a declining trend, there were no significant differences in the LORR time between the two groups (Fig. 4E), but the RORR time was markedly delayed in the mice receiving DAB compared with those receiving saline (Fig. 4F). The total percentage power of the EEG spectrum under 0.8% isoflurane exposure showed that the ratio of the δ band increased after DAB injection, and no significant differences in the other bands were found between the two groups (Fig. 4G–I). Hence, we concluded that blocking glycogenolysis by DAB has an anesthesia-promoting effect.

### Augmentation of Glycogenolysis Reduces EEG-δ Frequency, Prolongs Anesthesia Induction, and Facilitates Emergence from Anesthesia

**Fig. 5:**
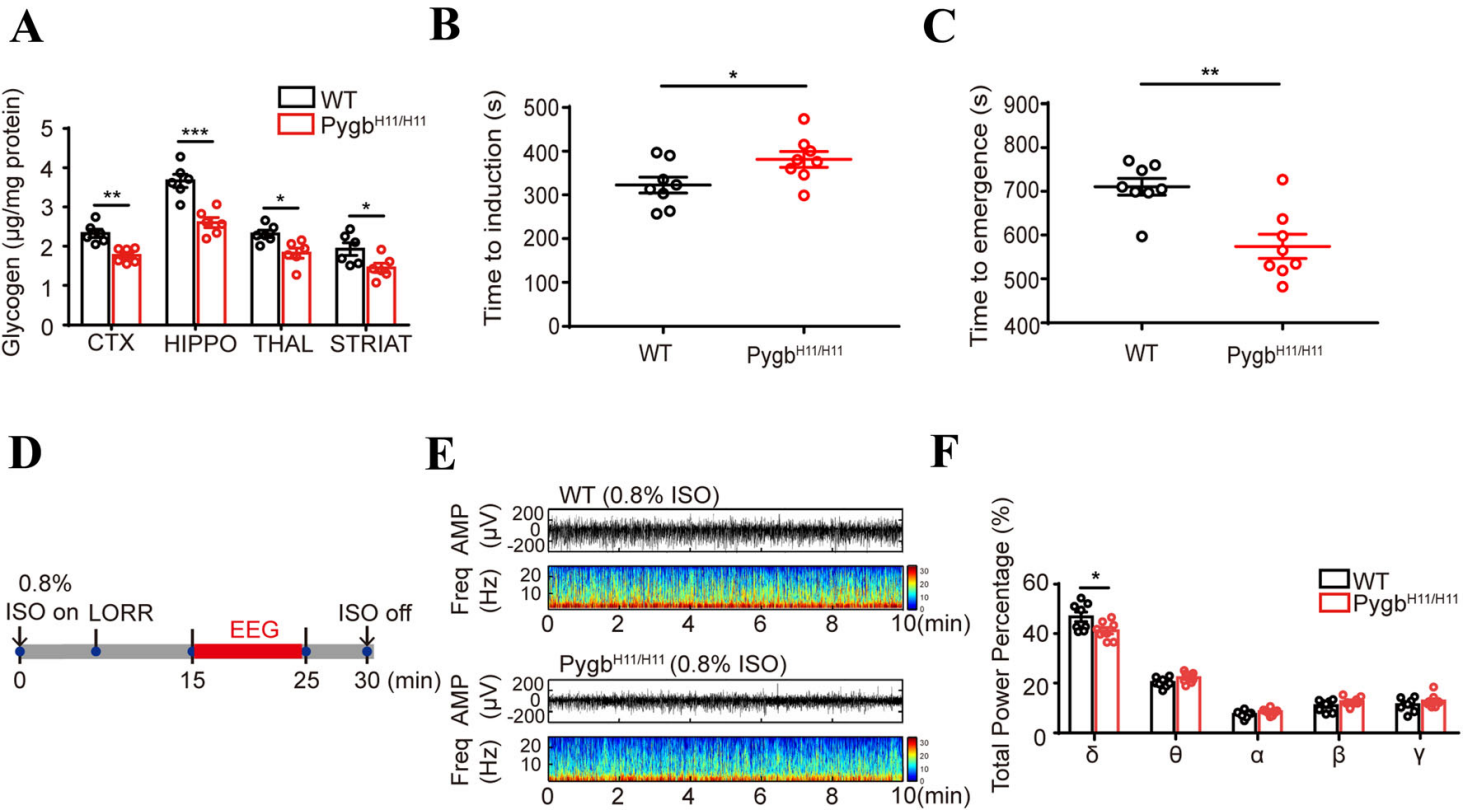
Pygb^H11/H11^ mice exhibit a decreased arousal time from isoflurane anesthesia. **A** Comparison of glycogen levels in several brain regions of PYGB^H11/H11^ mice and their WT littermates after exposure to isoflurane for 2 h (*n =* 6 per group). **B**, **C** The LORR (**B**) and RORR (**C**) times of PYGB^H11/H11^ and WT mice exposed to isoflurane for 2 h (*n* = 8 per group). **D** Protocol for recording EEG frequency in mice under 0.8% isoflurane anesthesia for 10 min, beginning 15 min after initial administration of isoflurane. **E** Representative EEG traces and heat maps. **F** Total percentage power of the EEG bands in mice under 0.8% isoflurane anesthesia (*n* = 8 per group). *P < 0.05, **P < 0.01, ***P < 0.001.

To further corroborate the role of glycogenolysis in isoflurane anesthesia, we generated GFAP-specific Pygb knock-in mice by using CRISPR/Cas-mediated genome editing to insert the “GFAP-mouse Pygb CDS-Poly A” cassette into the H11 locus on mouse chromosome 11 (Fig. S2A). Genotypes were identified by PCR, and homozygous Pygb^H11/H11^ mice (747 bp/747 bp) and WT littermates (519 bp/519 bp) were used in the experiments (Fig. S2B). Immunochemistry and immunoblotting were used to evaluate the gene-targeting efficiency of Pygb overexpression, both of which demonstrated a significant increase in PYGB expression compared to that in WT littermates (Fig. S2C, D). Under isoflurane anesthesia, the glycogen levels in the four assessed regions were markedly higher in Pygb^H11/H11^ mice than in WT littermates (Fig. 5A). Compared with WT littermates, Pygb^H11/H11^ mice exhibited an increased time to LORR (Fig. 5B) and a reduced time to RORR (Fig. 5C). EEG signals showed that the of δ-band ratio was significantly decreased. In addition, there were no significant differences in the other EEG bands between the two groups (Fig. 5D–F). Taken together, these results suggested that the enhanced capacity for glycogenolysis in Pygb^H11/H11^ mice leads to an arousal-promoting effect.

## **Discussion**

In the current study, we demonstrated that exposure to the inhalational anesthetic isoflurane increased the levels of glycogen in various brain regions: the CTX, HIPPO, THAL, and STRIAT. Decreased phosphorylation of GS at Ser641 and increased phosphorylation of GP at Ser15, which were accompanied by increased activity of the two rate-limiting enzymes in glycogen metabolism, occurred after 2 h of isoflurane anesthesia. Interventions targeting brain glycogen changed anesthesia-associated behaviors, such as the latency of the LORR and RORR and the EEG spectrum. We conclude that the availability of brain glycogen is critical in the regulation of recovery from isoflurane anesthesia.

Brain glycogen, which is regarded as an advantageous form for glucose storage in astrocytes [15], has been implicated in various physiological and pathological events, such as memory formation, sleep, aging, exercise, migraine, and stroke [23, 29–32]. Brain glycogen levels have also been proposed to increase during general anesthesia, and the degree of increase is related to both the depth and duration of general anesthesia and the type of anesthetic used [24–26]. However, whether brain glycogen can be manipulated to regulate the effects of anesthesia remains unknown. Here, we found that the glycogen levels in the CTX, HIPPO, THAL, and STRIAT mildly increased during isoflurane exposure in a time-dependent manner, in agreement with previous studies [26]. Furthermore, we refined the correlation between the changing pattern of brain glycogen levels and anesthetic status: the induction of, maintenance of, and emergence from anesthesia. We concluded that brain glycogen levels increase as anesthesia progresses and decrease as arousal progresses. Among the analyzed regions, the THAL showed strikingly sensitive changes in glycogen levels in response to the changes in anesthetic status, suggesting that glycogen in the THAL might play a distinctly important role in the regulation of anesthesia.

Since glycogen synthesis and degradation occur simultaneously, glycogen content varies depending upon the immediate energy needs of the tissue [33, 34]. Two rate-limiting enzymes, GS and GP, are involved in the regulation of glycogen metabolism [35]. According to our immunofluorescent staining results, GP is exclusively expressed in astrocytes, while GS is localized to both neurons and astrocytes in adult mice. The results are also supported by previous studies [36]. GS exists in two forms: the inactive phosphorylated form and active dephosphorylated form; its activity is controlled by glycogen synthase kinase-3 (GSK-3) and protein kinase A (PKA) [37]. GP, by contrast, exists in an active phosphorylated state and inactive dephosphorylated state; its activity in the brain is regulated by phosphorylase kinase (PhK) and the allosteric binding of AMP and glucose [38, 39]. Our previous study demonstrated that sevoflurane exposure induces the hyperphosphorylation of GSK-3β [40], resulting in a shift towards GS dephosphorylation. Here, we provide evidence that isoflurane exposure results in decreased phosphorylation of GS at Ser641 (p641-GS), in accordance with the increased GS activity found in the biochemical assay. Notably, isoflurane exposure also elicited hyperphosphorylation of GP at Ser15 (p15-GP) and increased GP activity in the CTX, THAL, and STRIAT. However, the levels of phosphorylated GP in the HIPPO showed no detectable change in our study. Additional studies are needed to clarify the heterogeneity of brain glycogen metabolism among brain regions.

The biosynthesis of brain glycogen depends heavily on the supply of blood glucose[41, 42], the levels of which tend to correlate with brain glycogen levels [43]. It has been reported that blood glucose levels are elevated after isoflurane anesthesia [44]. However, the relationship between blood glucose levels and brain glycogen under isoflurane anesthesia were still unclear. In this study, we investigated the pattern of blood glucose changes under different stages of isoflurane anesthesia (Fig. 2A, B), and found that the level began to increase significantly 30 min after 1.4% isoflurane anesthesia, then remained at the peak level (~14 mmol/L) for at least 4 h (Fig. S3A). The dynamic variations in blood glucose at different stages of isoflurane anesthesia were similar to those in brain glycogen shown in Fig. 2E (Fig. S3B). Considering the effect of brain glycogen and its underlying mechanism on anesthetic status is not totally understood, one possibility is that the effect might be induced by blood glucose levels [45]. Therefore, these levels were manipulated in mice by intraperitoneal injection of exogenous glucose (2 g/kg) or glucose starvation with extended fasting (24 h) under isoflurane exposure [46, 47] (Fig. S4A, B). The results showed that the glycogen levels in the CTX, HIPPO, THAL, and STRIAT were significantly increased after glucose injection and decreased after extended fasting (Fig. S4C), in accordance with previous studies [48]. Besides, a low blood glucose level significantly delayed the time to RORR, while a high blood glucose level did not change the time to RORR (Fig. S4D). Since our pharmacological intervention targeting glycogenolysis only affected RORR (Fig. 4E, F), and in the interest of the clinical value of arousal modulation [49], in this experiment, we paid more attention to the effects of blood glucose level on RORR rather than on LORR.We speculate that the underlying mechanism that the extended fasting-induced delayed RORR is associated with the abnormal metabolism and dysfunction of neuronal cells under conditions of hypoglycemia, such as mitochondrial damage and oxidative stress [50, 51]. In addition, one explanation why glucose injection did not affect the time to RORR is that isoflurane anesthesia itself induces hyperglycemia [44], additional glucose feeding might not change the arousal from anesthesia. The mechanism underlying the arousal-modulating effect of brain glycogen after general anesthesia deserves further investigation.

Glycogenolysis in astrocytes is strictly associated with the fate of glucose as described by the astrocyte–neuron lactate shuttle (ANLS) hypothesis, which states that astrocyte-derived lactate supports neuronal metabolism and plasticity [30, 52]. To date, controversies about the existence and significance of the ANLS continue [53]. The “energetic” explanation is that the glycogen stored in astrocytes is rapidly converted to pyruvate/lactate *via* glycolysis. As a predominant energy substrate, lactate is transferred from astrocytes to neurons and metabolized in the tricarboxylic acid cycle for ATP synthesis during functional activation [18]. The rates of oxidative phosphorylation and changes in ATP concentrations alter thesensitivity of *C. elegans* and rodents to volatile anesthetics [11, 54, 55].We found that DAB treatment resulted in significant amelioration of glycogenolysis and consequently induced an increase in the ratio of the δ band in the EEG spectrum and delayed emergence from anesthesia. By contrast, compared with WT littermates, transgenic Pygb^H11/H11^ mice showed a lengthened induction time and reduced emergence time from anesthesia and the δ ratio in the EEG spectrum. Besides, a low blood glucose level induced by 24 h fasting significantly delayed the time to RORR, accompanied by reduced brain glycogen levels. These consolidated results suggested that changes in energy levels, particularly those produced by glycogen degradation, modulate the effects of general anesthesia by isoflurane.

A previous study based on ^13^C magnetic resonance spectroscopy *in vivo* proposed that lactate, a product of glycogenolysis, might be used as an alternative metabolic substrate under thiopental anesthesia [56]. Here, we found increased GP activity in the CTX, THAL, and STRIAT after isoflurane exposure for 2 h, indicating that isoflurane has an energy metabolism pattern similar to that of thiopental anesthesia to some degree. We speculate that the elevated GP activity is attributable to a compensatory reaction following the elevated glycogen level. Additional studies, such as investigating the time course of GS and GP activity, might uncover the mechanism.

A main limitation of our study is that we did not evaluate metabolites of glycogenolysis, such as lactate and ATP. Cell-based experiments could be designed to explore the energy network, especially the ANLS hypothesis, between astrocytes and neurons in the future. In addition, since the levels of blood glucose in the glucose injection group were already decreased after 2 h of isoflurane exposure compared with the beginning of anesthesia (Fig. S4B), persistent perfusion or different doses of glucose should be used to determinethe effect of hyperglycemia on RORR under isoflurane anesthesia.

In conclusion, our results show that the dynamic variations in brain glycogen levels during general anesthesia are crucial to anesthesia–arousal modulation. Developing novel drugs targeting the degradation of brain glycogen may offer a promising strategy to modulate arousal during general anesthesia.

## Electronic supplementary material

Below is the link to the electronic supplementary material.

Supplementary material 1 (PDF 1189 kb)
