# Supplementary material for: Dynamic Variations in Brain Glycogen are Involved in Modulating Isoflurane Anesthesia in Mice
Source: Neurosci Bull. 2020 Oct 13;36(12):1513–23. doi: 10.1007/s12264-020-00587-3 (PMC7719152; doi:10.1007/s12264-020-00587-3)
Supplement: Supplementary file 1 — Supplementary material 1 (PDF 1189 kb) [file 12264_2020_587_MOESM1_ESM.pdf]

Supplemental Materials

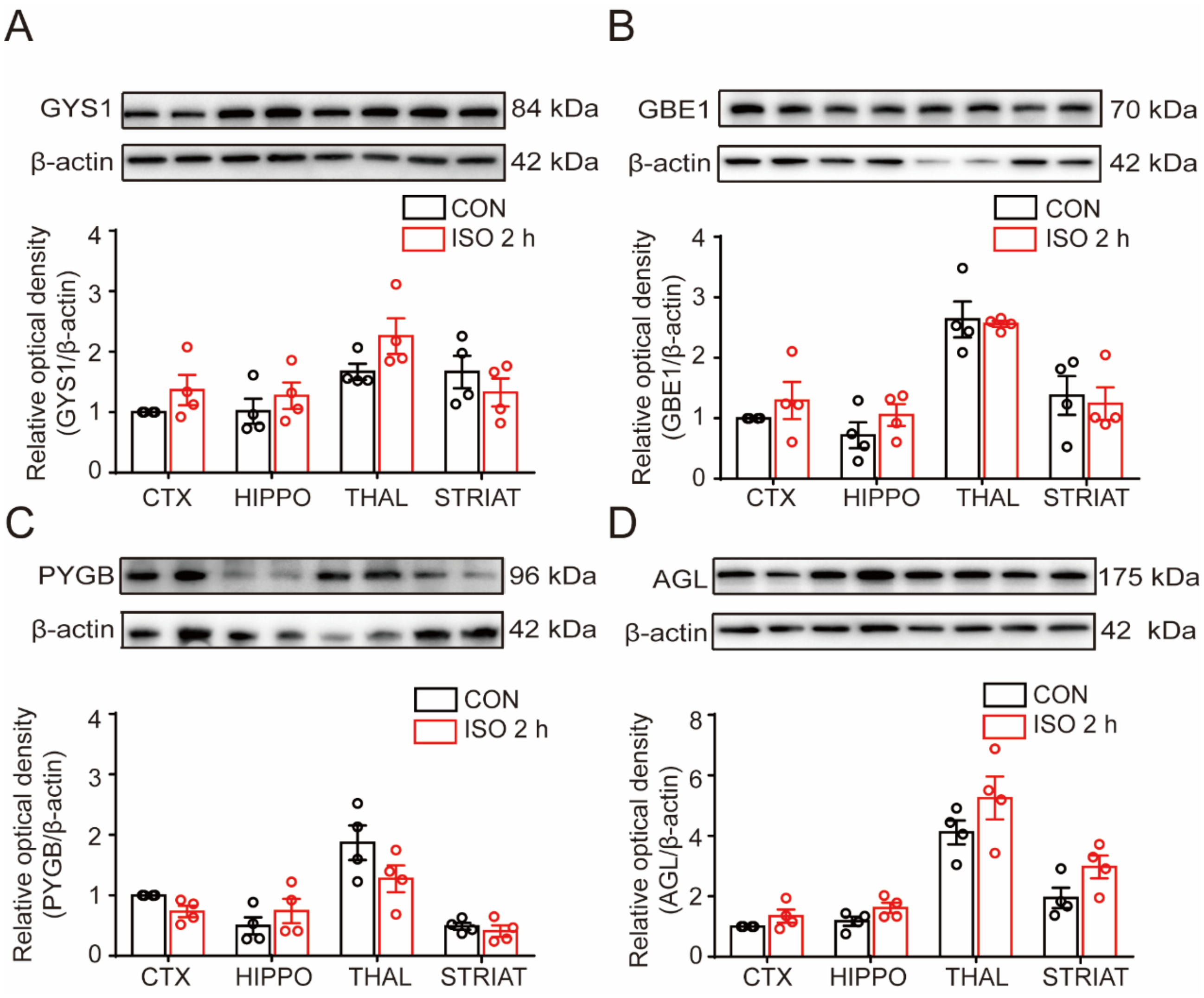

Fig. S1 Effects of isoflurane exposure on key enzymes of glycogen metabolism. A–D Immunoblots and statistics showing the levels of GYS1 (**A**), GBE1 (**B**), PYGB (**C**), and AGL (**D**) in the CTX, HIPPO, THAL, and STRIAT of mice in the presence or absence of isoflurane (*n*= 4 per group). CON, oxygen control; ISO 2 h, isoflurane exposure for 2 h; CTX, cortex; HIPPO, hippocampus; THAL, thalamus; STRIAT, striatum.

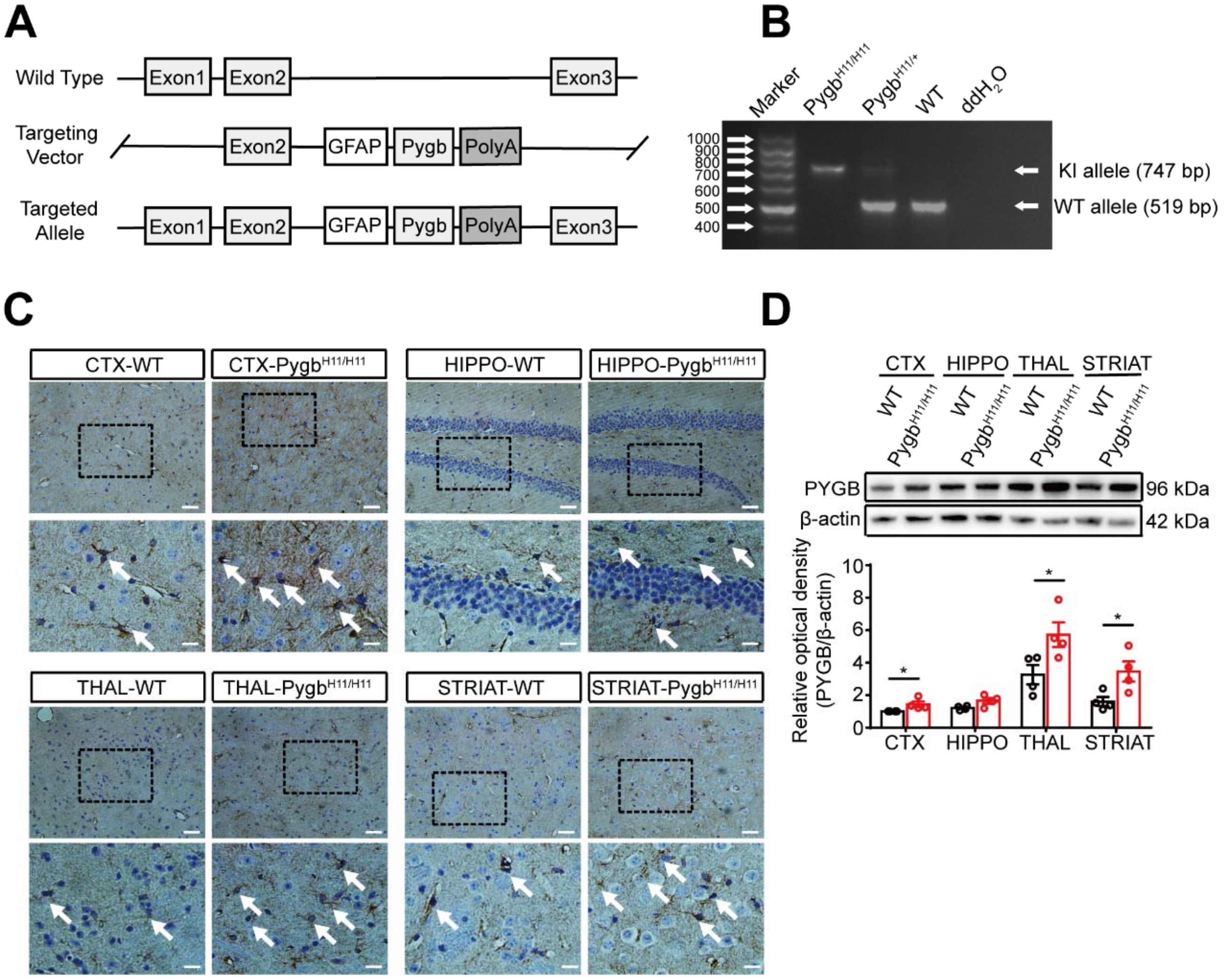

Fig. S2 Upr egulation of PYGB in Pygb $^{H11/H11}$ mice. A Schematic overview of the CRISPR/Cas-mediated genome strategy for creating GFAP-specific Pygb knock-in mice. **B** PCR bands of Pygb $^{H11/H11}$, Pygb$^{H11/+}$ and WT mice. Homozygous knock-in: 747 bp/747 bp; heterozygotes: 747 bp/519 bp; WT: 519 bp/519 bp. **C**, **D** Representative immunochemistry (**C**) and immunoblotting (**D**) to confirm GP overexpression (n= 4 per group). Scale bars, 50 μm in upper panels, 20 μm in lower panels; *P <0.05.

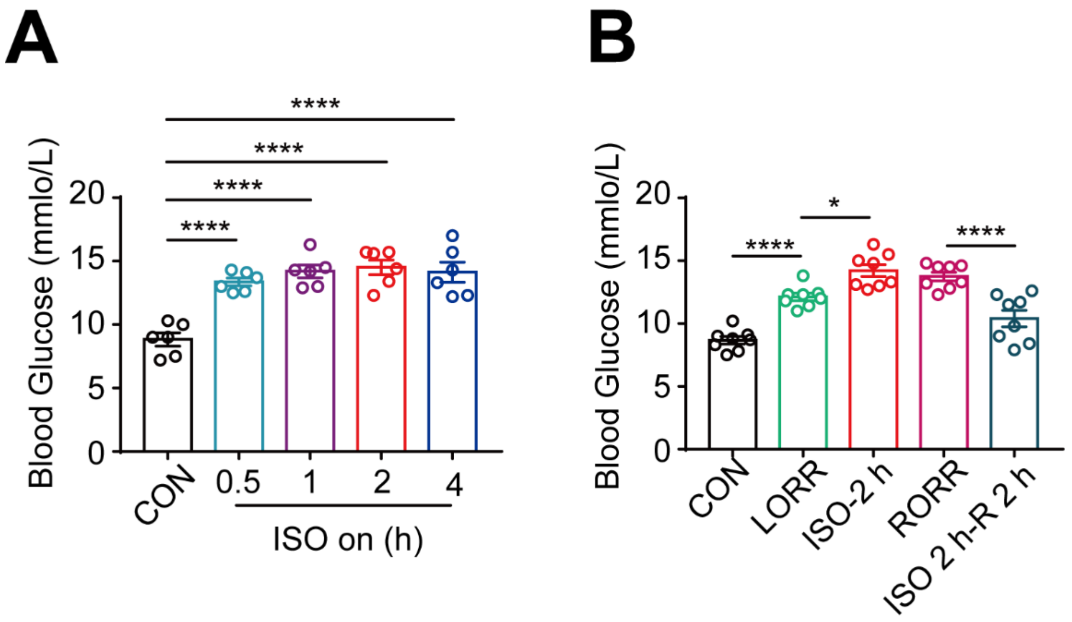

Fig. S3 Dynamic variations in blood glucose levels under isoflurane anesthesia. A Blood glucose concentrations increase after isoflurane exposure (*n*= 6 per group). **B** Patterns of changes in blood glucose concentrations after isoflurane anesthesia (*n*= 8 per group; **P* <0.05, *****P* <0.0001).

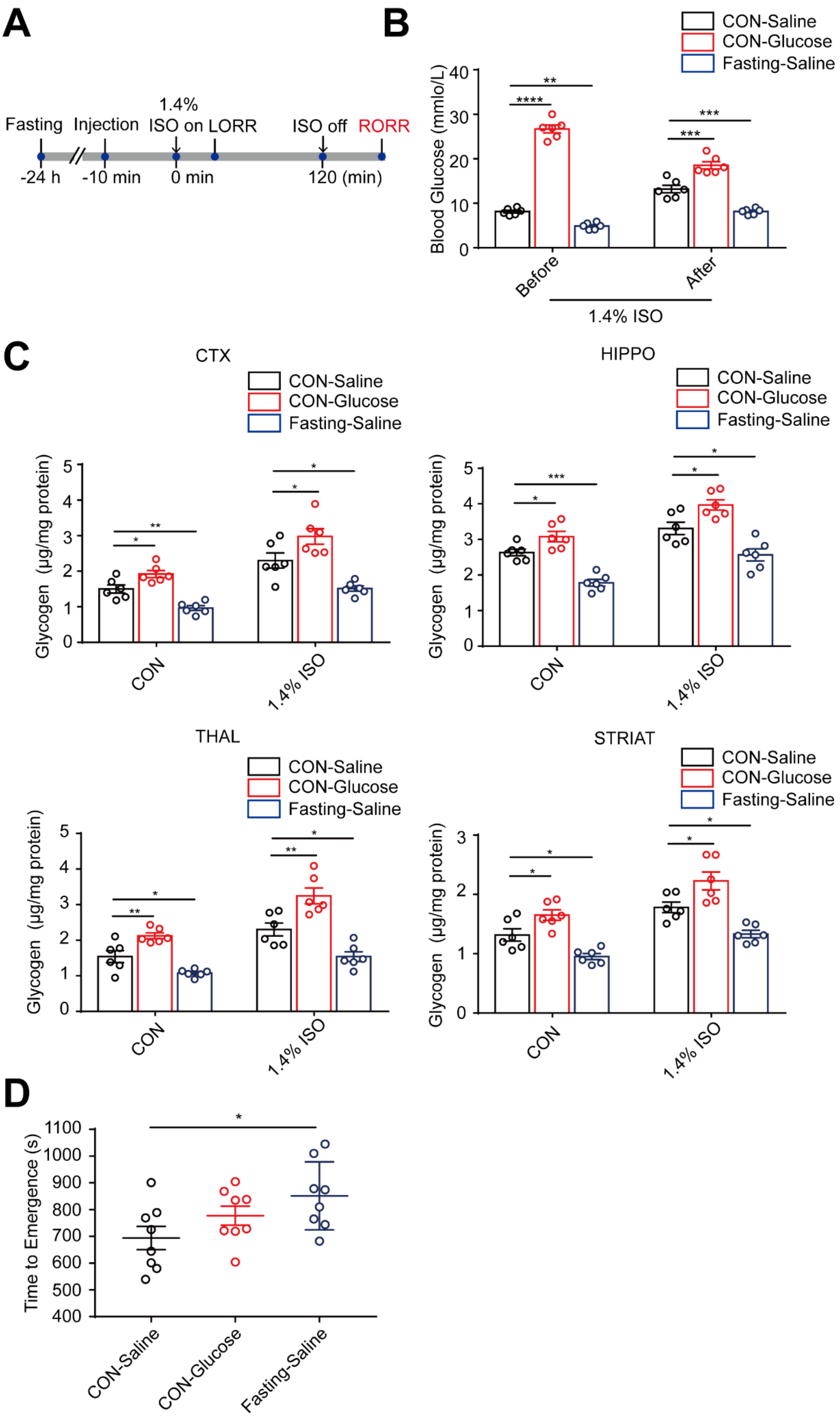

A
Fasting
Injection
1.4%
ISO on LORR
ISO off
RORR
-24 h
-10 min
0 min
120 (min)
B
CON-Saline
CON-Glucose
Fasting-Saline
Blood Glucose (mmlo/L)
Before
After
1.4% ISO
C
CTX
HIPPO
THAL
STRIAT
Glycogen (μg/mg protein)
CON
1.4% ISO
D
Time to Emergence (s)
CON-Saline
CON-Glucose
Fasting-Saline

Fig. S4 Blood-borne glucose affects brain glycogen levels and anesthesia–arousal. A Protocol for recording the RORR time under 1.4% isoflurane anesthesia after blood glucose regulation. **B** Changes in blood glucose levels before and after 2 h of isoflurane anesthesia (*n*= 6 per group). **C** Brain glycogen levels in selected brain regions of mice with or without 2 h of isoflurane anesthesia after blood glucose regulation (*n*= 6 per group). **D** RORR time after 2 h of isoflurane anesthesia (*n*= 8 per group). *P <0.05, **P <0.01, ***P <0.001, ****P <0.0001.

Table S1 Sources of antibodies

**Antibody Target**	**Source**	**Application**	**Dilution**	**Identifier**
AGL	Abcam	IB	1:1000	Ab133720
β-actin	Abcam	IB	1:5000	Ab119716
GBE1	Abcam	IB	1:1000	Ab180596
GYS1	Abcam	IB/IF	1:1000/1:25	Ab40867
phospho-GS (Ser641)	CST	IB	1:500	#3891
PYGB	Abcam	IB	1:1000	Ab154969
PYGB	ATLAS	IF/IHC	1:50/1:500	HPA031067
phospho-PYGB (Ser15)	Gene Create Biotech	IB	1:250	Customized antibody

GFAP	GeneTex	IF	1:500	GTX85454
NeuN	Millipore	IF	1:200	MAB377
Iba-1	Abcam	IF	1:300	Ab5076
Anti-rabbit (Fluor 488)	TFS	IF	1:500	A32731
Anti-chicken (Fluor 594)	TFS	IF	1:500	A32759
Anti-mouse (Fluor 594)	TFS	IF	1:500	A32744
Anti-goat (Fluor 594)	TFS	IF	1:500	A11058
IgG H&L (HRP)	Abcam	IB	1:5000	Ab6721

CST, Cell Signaling Technology; TFS, Thermo Fisher Scientific; IB, immunoblotting; IHC, immunohistochemistry; IF, immunofluorescence
